# Performance of the Digital Dietary Assessment Tool MyFoodRepo

**DOI:** 10.3390/nu14030635

**Published:** 2022-02-01

**Authors:** Claire Zuppinger, Patrick Taffé, Gerrit Burger, Wafa Badran-Amstutz, Tapio Niemi, Clémence Cornuz, Fabiën N. Belle, Angeline Chatelan, Muriel Paclet Lafaille, Murielle Bochud, Semira Gonseth Nusslé

**Affiliations:** 1 Center for Primary Care and Public Health (Unisanté), University of Lausanne, 1010 Lausanne, Switzerland; patrick.taffe@unisante.ch (P.T.); gerrit.burger@student.uni-tuebingen.de (G.B.); wafa.badran-amstutz@unisante.ch (W.B.-A.); tapio.niemi@unisante.ch (T.N.); clemence.cornuz@vd.ch (C.C.); fabien.belle@ispm.unibe.ch (F.N.B.); murielle.bochud@unisante.ch (M.B.); semira.gonseth-nussle@unisante.ch (S.G.N.); 2 Institute of Social and Preventive Medicine (ISPM), University of Bern, 3012 Bern, Switzerland; angeline.chatelan@hesge.ch; 3 Department of Nutrition and Dietetics, School of Health Sciences (HEdS-GE), University of Applied Sciences and Arts Western Switzerland (HES-SO), 1227 Carouge, Switzerland; 4 Department of Endocrinology, Diabetology and Metabolism, Lausanne University Hospital (CHUV), 1011 Lausanne, Switzerland; muriel.lafaille@chuv.ch

**Keywords:** dietary assessment, accuracy, validation, food intake, diet, mobile food record, app

## Abstract

Digital dietary assessment devices could help overcome the limitations of traditional tools to assess dietary intake in clinical and/or epidemiological studies. We evaluated the accuracy of the automated dietary app MyFoodRepo (MFR) against controlled reference values from weighted food diaries (WFD). MFR’s capability to identify, classify and analyze the content of 189 different records was assessed using Cohen and uniform kappa coefficients and linear regressions. MFR identified 98.0% ± 1.5 of all edible components and was not affected by increasing numbers of ingredients. Linear regression analysis showed wide limits of agreement between MFR and WFD methods to estimate energy, carbohydrates, fat, proteins, fiber and alcohol contents of all records and a constant overestimation of proteins, likely reflecting the overestimation of portion sizes for meat, fish and seafood. The MFR mean portion size error was 9.2% ± 48.1 with individual errors ranging between −88.5% and +242.5% compared to true values. Beverages were impacted by the app’s difficulty in correctly identifying the nature of liquids (41.9% ± 17.7 of composed beverages correctly classified). Fair estimations of portion size by MFR, along with its strong segmentation and classification capabilities, resulted in a generally good agreement between MFR and WFD which would be suited for the identification of dietary patterns, eating habits and regime types.

## 1. Introduction

Although diet is recognized as a large contributor to the onset and etiology of non-communicable diseases, its valid and reliable measurement in clinical and epidemiological studies remains a challenge, mainly because of its reliance on self-reported information and the lack of accessible tools to collect good quality information. Conventional dietary assessment tools are either: of good scientific quality but involve high implementation costs (24-h recall) and substantial commitment from the participants (dietary records); or are easily implemented but lack accuracy and precision (food frequency questionnaires) [1].

Digital measurement devices can help overcome the limitations of conventional dietary assessment tools and provide a cost-effective way to simplify and scale up nutritional data collection. Such devices were shown to increase user acceptance while providing valuable real-time food intake data [2,3,4], and have the potential to eliminate participant burden linked to portion size estimation [5]. Digital image capture of foods is further facilitated by the distribution of mobile phones and the population’s familiarity with this technology. In 2017, more than 325,000 health mobile applications were available via major app stores all over the world [6]. The majority of available mobile dieting apps are designed to support behavioral changes and either have not been validated for use in research or lack accuracy [7,8,9,10]. Although digital dietary assessment tools employed in research overcome these limitations, they often rely on more cumbersome participation from users; for example, necessitating specific experimental settings [11], requiring participants to wear impractical gear (e.g., chest-worn camera) [12,13,14] or manually select dishes and estimate portion sizes [2,15,16,17]. The accuracy of these tools relies on the comprehensiveness and quality of their underlying nutrition databases, whose continuous update is a challenge [3,10]. The relevance of emerging digital dietary assessment tools for epidemiological research is also made difficult by the scarcity of information relative to the tools’ development process, the large variation in intake calculations and the differences in methodologies employed amongst validation studies [10]. Validation studies rarely assess all the different stages of dietary recognition: (1) segmentation (i.e., the ability of a tool to recognize where the different edible components of an image find themselves); (2) classification (i.e., the ability to correctly identify what the content of each segment is); (3) portion size estimation and (4) energy and macronutrient calculation. Whereas some researchers investigating digital tools mainly focus on the segmentation and classification of food components—as is the case for DietCam [18]—other validation studies—e.g., for e-Ca [19] or mFR [20]—concentrate on determining weight error and related energy and macronutrient intakes. In most validation studies—amongst which the performance analysis of the digital dietary assessment tools Keenoa [9], PIQNIQ [2], EaT [21] and Bridge2U [22]—the accuracy of energy and macronutrient content is the sole endpoint investigated. One exception is Snap-n-Eat, a mobile phone recognition system able to perform automatic segmentation and classification of foods and allowing subsequent weight estimation and energy and macronutrient content calculation, but this app has not yet been validated [23].

The aforementioned tools often present low to moderate levels of accuracy [4,24], showing wide limits of agreement compared to established dietary methods [9,21,22]. Newly developed digital dietary assessment tools need to be compared against a reference method (e.g., weighted food diaries over several days) and assessed among participants with similar dietary habits as the population of interest, which is not systematically the case.

In this context, we aimed to assess the accuracy of the automated dietary assessment device MyFoodRepo, by investigating its capability to identify, classify, and estimate portion sizes and determine the macronutrient content of diet against reference values from weighted food diaries.

## 2. Materials and Methods

MyFoodRepo (MFR) is a mobile application developed by the team of Prof. Marcel Salathé (Digital Epidemiology Lab-École Polytechnique Fédérale de Lausanne), which can be used to track food consumption from pictures of meals and beverages or from scanned food products’ barcodes [25]. The app does not require any fiduciary marker for image recognition and its algorithm, based on thousands of images (May 2021), uses artificial intelligence for image content analysis. The system incorporates an annotation interface, which allows textual conversation between MFR app users and a human reviewer from the MFR app developers’ team.

Three researchers conducted the present validation study, using the photography and barcode scanning features of MFR mobile app to record foods and beverages. MFR was evaluated on four different criteria: (1) segmentation (the ability to accurately differentiate the distinct edible components of a record, for example discerning the presence of a beige-colored segment from a red-colored segment in a plate while leaving aside the background and cutlery); (2) classification,(the ability to correctly identify the content of each detected segment, for example, identifying that the beige-colored segment is pasta and that the red-colored segment is Bolognese sauce); (3) portion size estimation (the accuracy of weight estimates for each detected and exactly classified segment); (4) overall performance (the accuracy and agreement of energy and macronutrient estimates compared to weighted food diaries).

### 2.1. Data Collection

Data collection extended from September to December 2019. We aimed at gathering a minimum of 180 records—1 record defined as either 1 photograph or 1 scan entered into MFR—distributed as such: 60 composite foods, made up of ≥3 segments; 60 simple foods, made up of 1–2 segment(s); 30 composite beverages, made up of ≥2 segments; 30 simple beverages, made up of 1 segment only. For foods, a segment may refer to a single ingredient (e.g., carrots) or mixed ingredients that form a unified item to be recognized by MFR (e.g., ratatouille). For beverages, a segment refers to a single ingredient (e.g., tea). Records were arbitrarily selected by the researchers and did not represent daily intake. Industrial processed foods with a barcode were directly scanned into the app.

#### 2.1.1. Controlled Values Measured from the Weighted Food Diaries

To produce controlled values, we created, tested and optimized food diaries with the advice of a registered dietician. We entered each record into the food diaries. Weight and nutritional values from barcoded products were directly transcribed from their respective packaging and nutrition labels. Ingredients and complete segments from photographed records were carefully weighed and described. For cooked or mixed items, we noted the precise recipe.

Data from the weighted food diaries were analyzed by the dietician using the software PRODI 6.5 Swiss (Nutri-Science GmbH, Hausach, Germany) and food composition databases, resulting in nutritional values being retrieved from the Swiss Food Composition Database [26], the French Food Composition Database [27] and the German Nutrient Database [28] to obtain energy and macronutrient content data on all records.

We additionally classified all segments into 37 food types (Table S1). Segments made up of mixed ingredients were classified according to the ingredient with the highest calorific content (e.g., potato gratin classified into “Tubers”). Food types were further coded into 23 food groups and again into 7 food categories, corresponding to categories of the Swiss food pyramid [29].

#### 2.1.2. Measurements Made by MFR

For each record entered into the weighted food diaries, a new picture or scan was saved into MFR. Records were processed by the MFR algorithm, and curated by the MFR app developers, who were able to ask for clarifications about the entered records via the built-in annotation interface of the app. To test MFR’s ability to recognize and analyze food content, the researchers were instructed not to leave any spontaneous descriptions about the content of the pictured foods in the app’s annotation interface. However, researchers were allowed to answer questions posed by the MFR app developers (e.g., “Did you put sugar in the tea?”; “Is it beef or veal in the picture?”). All MFR app developers performed their tasks while being blind to the study, since they did not know if they had interactions with researchers from this validation study or participants from other ongoing studies.

MFR draws the nutritional values of food and beverages from the Swiss Food Composition Database [26] and the French Food Composition Table, Ciqual [27]. Nutritional values of barcode scanned records are extracted from MFR’s community-driven associated database, Open Food Repo [30], whose members can add and correct information from nutrition labels.

The researchers obtained the data extracted from MFR by the app development team on 17 February 2021 with details on date and time of collection, name, weight and/or volume, energy and macronutrient content of each detected segment contained in the records. The researchers consequently listed all segments identified by the MFR app, and classified them into food types, groups and categories.

### 2.2. Data Analysis

#### 2.2.1. Segmentation

Segments (i.e., different components of a record) correctly identified by MFR were coded as found segments (F); overlooked segments were coded as omissions (O); additional segments erroneously identified by MFR and not actually present on a record were coded as intrusions (I). The segmentation percentage of accuracy was then calculated by the number of found, omitted and intruded segments, respectively, over the total number of original segments in the weighted food diaries.

#### 2.2.2. Classification

MFR naming of each found segment was compared to the corresponding true segment designation. MFR’s classification performance was assessed by describing each match as exact match, close match, far match or mismatch according to the following criteria: exact match (E): MFR segment belongs to the correct food type and/or is labeled after a product meaning the exact same thing (e.g., cherry tomatoes vs. tomatoes); close match (C): MFR segment belongs to the correct food type but its naming is either too generic, not specific enough, refers to a slightly different product (e.g., beef vs. veal) or contains an overlooked ingredient within (e.g., tea with added sugar vs. tea); far match (F): MFR segment belongs to the wrong food type but to the correct food group (e.g., pasta vs. rice); mismatch (M): MFR segment belongs to the wrong food type and the wrong food group (e.g., carrot vs. potato).

The classification accuracy was calculated as the percentage of exact, close, far and mismatches among the total number of found segments. Proportion differences between MFR and controlled values were tested using Fisher’s exact test. 

To evaluate MFR categorization performance at different levels of granularity, it was assessed by main food categories, food groups and food types using the Cohen kappa as a reliability indicator and the uniform kappa coefficient as an agreement indicator, specificity and sensitivity calculation. As inter-rater agreement between two researchers judging MFR segmentation and classification was high for 30 random records (uniform kappa Ku = 1 [95% confidence interval: 1;1] and Ku = 0.744 [0.607;0.843], respectively [31]), one researcher could proceed with coding the remaining records.

#### 2.2.3. Portion Size Estimation

The weight error (difference between MFR weight estimation and true weight) was determined for each exactly classified segment. Mean weight, mean error, and mean absolute error were then calculated per food type, food group and food category and mean errors plotted into boxplots. The mean differences between true and estimated values were assessed with paired *t*-tests. Two-sided *p*-values ≤ 0.05 were considered significant. Finally, the accuracy of portion size estimates was assessed dividing the mean estimated weight by the mean true weight for each food type, group or category.

#### 2.2.4. Overall Performance for Energy and Macronutrient Content

To assess the agreement between the two methods, linear regression analysis was performed for energy and macronutrient estimates (fat, carbohydrates, proteins, fiber, alcohol). Linear regression was preferred (with MFR measurement as the dependent variable and weighted food diaries’ measurements as the independent variable) to the commonly used Bland–Altman method, as the latter was shown to provide biased results when one of the two measurement methods has negligible measurement error [32]. Therefore, under the assumption that weighted food diaries correspond to an unbiased gold standard with negligible measurement error, we estimated the differential and proportional bias from the app, by regressing MFR measurements as a function of the controlled values. The 95% limits of agreement were then calculated by modeling the measurements’ heteroscedasticity from the app [33]. To allow for comparison and give a general idea of the MFR data dispersion, we additionally calculated the coefficient of variation of MFR at different controlled values (25th percentile, median, 75th percentile) as well as the mean coefficient of variations ($\bar{C\upsilon }$) for energy and macronutrients.

## 3. Results

In total, 189 records were collected (63 composite foods, 63 simple foods, 30 composite beverages, 33 simple beverages). Among all records, 174 (92%) were recorded by photography, while 15 (8%) were barcode scanned. For practical reasons, only simple foods and simple beverages benefited from the scan feature in this study. Clarifications were demanded by the MFR app developers via the annotation interface in 43% (*n* = 81) of all cases but exclusively for dishes and beverages recorded by photography (Figure S1).

Four probable weight transcription errors resulting in unrealistic entries in the food diaries were considered as outliers and removed from the portion size and overall performance analyses.

### 3.1. Segmentation

From 352 total true segments, MFR found 98.0%, omitted 2.0%, and intruded 1.4% (Table 1). Simple beverages had a 100% segmentation success rate and composite beverages had only one omission with the oversight of the decorative candy on a cocktail. Among food records, the app omitted six segments: capers, horseradish sauce, pasta sauce, vinegar, dried tomatoes and red peppers in vegetable mix, respectively. The five intrusions, all from foods, corresponded to the erroneous addition of salad dressing on salads.

The differences between composite and simple records were not significant, suggesting that MFR segmentation performance did not decrease with increasing complexity of the records and number of ingredients.

### 3.2. Classification

Among all 345 segments found by MFR, 87.5% ± 3.5 were classified as an exact match, 8.4% ± 3.0 as a close match, 1.2% ± 1.1 as a far match and 2.9% ± 1.8 as a mismatch (Table 2).

Scanned records showed perfect classification accuracy, with 100% of segments classified as an exact match.

The best results were found in records where clarifications had been demanded by the MFR app developers via the built-in annotation interface, compared to records where no clarification had been asked, with a slightly higher percentage of exact and close matches (98.4% ± 1.8 vs. 93.0% ± 4.0) and a lower percentage of far matches and mismatches (1.6% ± 1.8 vs. 7.0% ± 4.0) (*p* = 0.01339).

Simple records showed strong results with only two mismatches within simple beverages and three close matches within simple foods, the remaining simple records being classified as an exact match. Composite records presented more mitigated outcomes. MFR performed the poorest among composite beverages with only 41.9% (±17.7) exact matches. Close matches within composite beverages were mainly due to the omission of milk and/or sugar in hot beverages and the overly generic description of cocktails, whereas mismatches resulted from the erroneous classification of alcoholic beverages as soft drinks and soft drinks as non-alcoholic, non-sweetened beverages.

This influenced MFR classification results among food groups (Table 3), with the lowest sensitivity percentages observed among “non-alcoholic sweetened beverages” (75%) and “alcoholic beverages” (76.2%). Apart from small reductions in the food groups “sweeteners” (83.3%), “fats & oils” (85.7%) and “potatoes, legumes and beans” (90.5%), sensitivity percentages were otherwise high.

Uniform kappa results were high among food groups, laying between 0.971 [0.936;0.994] and 1 [1;1] and showing a strong classification agreement between MFR and controlled values. The Cohen kappa results, which measure reliability between the two methods, were prone to more fluctuation due to their high dependence to the number of segments in each discriminated food group.

Globally, classification reliability and agreement of MFR compared to controlled values were nevertheless high in all three levels of classification granularity: food categories (0.963), food groups (0.9554) and food types (0.9559) (Table 4).

Cohen’s kappa, uniform kappa, sensitivity and specificity by food types and food categories can be found in the Supplementary Materials (Tables S2 and S3).

### 3.3. Portion Size Estimation

The mean true weight of all exactly classified segments (*n* = 302) was 116.8 g ± 92.0, whereas mean estimated weight was 114.4 g ± 83.0 (*p* = 0.424), with a mean error of −2.4 g ± 51.8. Nevertheless, mean absolute error was 32.8% and the range of percentage error fluctuated between −88.5% and 242.5% of true weight.

**Figure 1:**
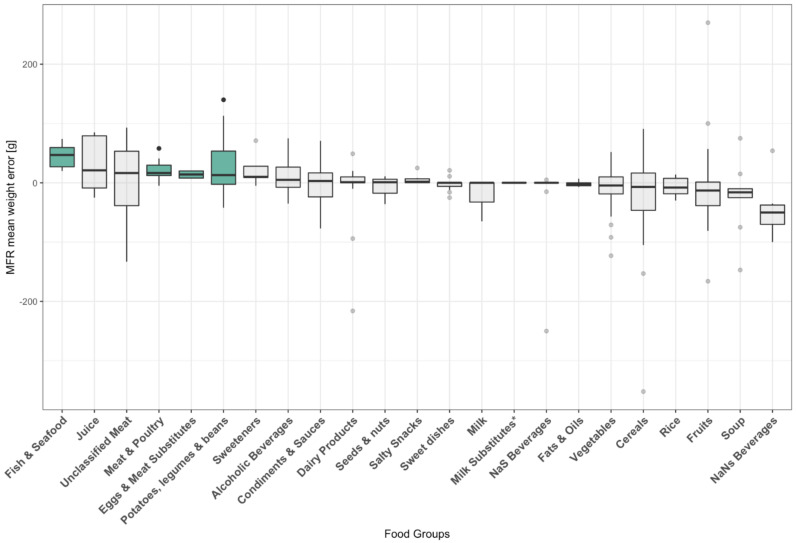
Mean weight errors per food group (NaNs: non-alcoholic non-sweetened; NaS: Non-alcoholic sweetened). Boxplots give median, interquartile range (IQR) and maximum 1.5 IQR. Colored boxplots indicate significant mean differences between estimated and true values (two-sided *p*-value ≤ 0.05). Four weight transcription errors resulting from unrealistic entries in the food diaries were removed from portion size analysis (not shown). * Only one observation in the “milk substitutes” food group.

Among all 23 food groups, half presented a mean absolute error between 25% and 50%. “Milk and milk-based beverages”, as well as “Non-alcoholic sweetened beverages” had a mean absolute error below 10%. On the other hand, “Fats & oils”, “Sweeteners” and “Condiments & sauces” showed a mean absolute error over 50%. As depicted in Figure 1, MFR significantly overestimated weight for “Meat & Poultry” (*p* = 0.0001), “Fish & Seafood” (*p* = 0.004), “Eggs & meat substitutes” (*p* = 0.027) and “Potatoes, legumes and Beans” (*p* = 0.023).

No significant differences were observed between estimated and true mean weight at the food category level.

MFR portion size estimation performance calculated by food types, food groups and food categories can be accessed in the Supplementary Materials (Tables S4–S6).

### 3.4. Overall Performance for Energy and Macronutrient Content

**Figure 2:**
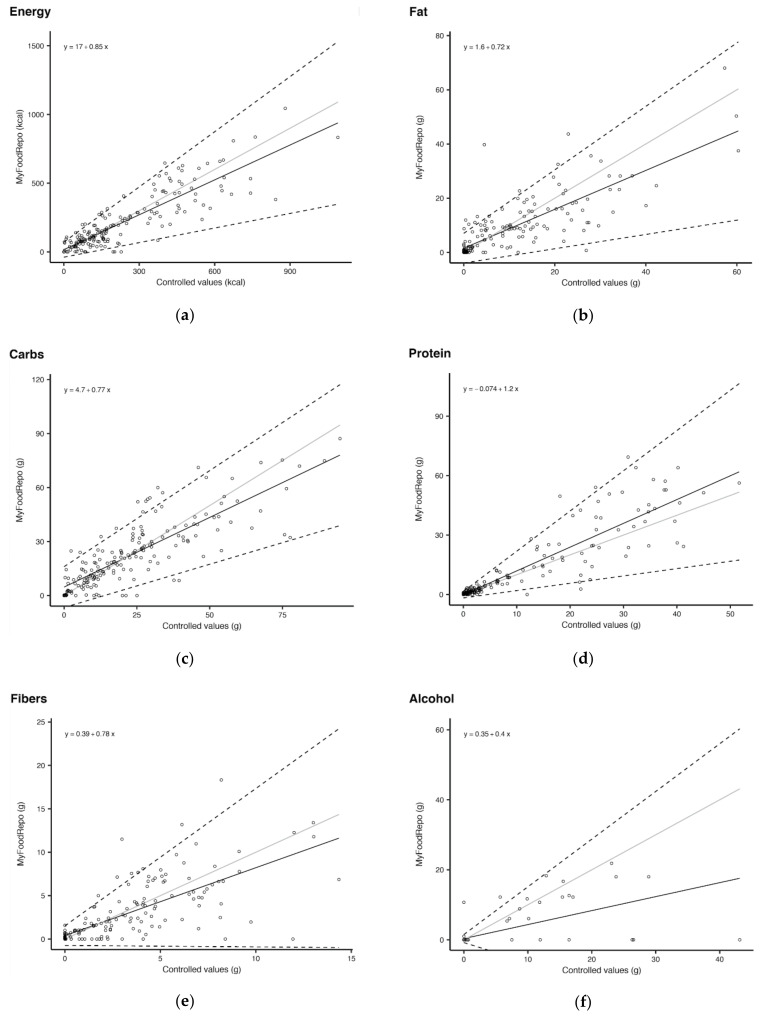
Overall performance for energy and macronutrient content: Linear regression of MFR versus controlled values for all found records, for content of (**a**) energy; (**b**) fat; (**c**) carbohydrates; (**d**) protein; (**e**) fiber and (**f**) alcohol. (Black line: linear regression line; dotted line: 95% limits of agreement; grey line: y = x).

The overall performance analysis included all 185 records. The linear regression performed on MFR measurements versus controlled values from the food diaries show an overestimation tendency by the app at small true values of energy and macronutrients and an underestimation tendency at higher true values (Figure 2). The y intercept was 113.3 kcal for energy, 5.7 g for fat, 20.4 g for carbohydrates, 1.8 g for fibers, and 0.6 g for alcohol.

Only the linear regression line for protein content fell above the 1:1 line, indicating a systematic overestimation of proteins by MFR.

For alcohol, note that the lower confidence line crossed the zero line. This happens because the 95% limits of agreement were built based on the Wald method with no transformation, indicating that the variance of the measurement errors is very large, in turn showing that the agreement is extremely poor.

The coefficients of variation (Cυ) and mean coefficients of variation ($\bar{C\upsilon }$) for energy and macronutrients of all records also suggest important levels of dispersion from MFR estimates (Table 5). As per the coefficients of variation (Cυ) calculated at the 25th percentile, median and 75th percentile of true values, MFR’s accuracy increased with increasing true energy and macronutrient content, meaning that the dispersion was higher for small quantities. The Cυ at the 25th percentile were particularly high for protein (1.96), alcohol (1.70), fat (1.68) and fibers (1.47). Fibers and alcohol had the highest $\bar{C\upsilon }$, whereas carbohydrates had the lowest $\bar{C\upsilon }$.

The mean coefficients of variation were higher for beverages compared to food records, except for alcohol and to a lesser extent, carbohydrates (Table 6). The higher alcohol $\bar{C\upsilon }$ in foods comes from the unaccounted alcohol content in some sauce recipes, showing MFR’s inability to identify sauce composition. Beverages $\bar{C\upsilon }$ negatively affected $\bar{C\upsilon }$ of all records, especially for fat and fibers.

Linear regression figures for food and beverages separately can be found in the Supplementary Materials (Figures S2 and S3).

## 4. Discussion

The purpose of this study was to assess the accuracy of the smartphone application MFR against weighted food diaries, which are currently considered the gold standard for dietary assessment. To our knowledge, this is the first study validating an automated digital dietary assessment tool by distinctly examining its different stages of food and beverage recognition, namely segmentation, classification, portion size estimation and energy and macronutrient content calculation.

Compared to most of its digital dietary assessment counterparts used in research, MFR requires minimal user input to record diet. While other digital dietary assessment tools require preliminary groundwork—for instance necessitating specific experimental settings with a fixed background [11]—others involve a fiduciary marker to be placed on the image [15,16,17] or for the users to delineate segments themselves in their specific tool [34,35]. Despite only relying on a smartphone camera, MFR showed strong segmentation capacity, identifying 98.0% of all segments present. This did not only include visible items, as for similar technologies [23], but also blended or mixed segments. Additionally, segmentation accuracy did not significantly decrease for complex records, unlike observations made by the automated dietary tool, DietCam [5,18].

MFR also performed generally well to classify found segments into food types, food groups and food categories. Scanned records showed perfect segmentation and classification results, bypassing the well-reported reduction in accuracy and reliability of dietary assessment tools associated with non-exhaustive databases [3,10]. Scanned items in MFR are indeed directly associated with the Open Food Repo database [30], which currently gathers more than 370,000 barcoded products sold in Switzerland. The database is open-access and user-enriched, ensuring its continuous update and alignment with population dietary habits. Uniform kappas over 0.958 indicated a good classification agreement between methods, at all levels of granularity. Percentages of exact matches exceeded 90% for composite foods, simple foods, and simple beverages, but only reached 41.9% for composite beverages, which could partially explain the large coefficients of dispersion for energy and macronutrients among beverages. “Alcoholic beverages”, “non-alcoholic sweetened beverages” and “non-sweetened non-alcoholic beverages” were classified interchangeably and additions of milk and/or sugar in tea and coffee were often overlooked.

Unlike MFR, many digital dietary assessment tools used in studies rely on user participation for portion size estimation, either via a portion size selector [2,19] or a complementary portion size booklet [22]. Portion size estimation relying on the capture of a single image was proven to reduce user burden as automated estimations are not affected by the user’s lack of knowledge about quantities [36,37]. Photography can also decrease data collection time and participant’s disturbances in complex settings such as school cafeterias [11], and facilitate study implementation in environments with lower health and nutrition literacy or language barriers [38,39]. MFR would nonetheless benefit from more precise estimations of portion sizes. Although the global mean portion size error was −2.4 g ± 51.8 or 9.2% ± 48.1%, the error range produced by individual errors varying between −88.5% and +242.5% of true weight was wider than observed in the existing literature. In comparison, the electronic mobile-based food record e-Ca showed a mean error of 3% with errors ranging between −38% and +130% of true weights across 20 food and beverages displayed in a controlled setting [19], whereas the MFR app, developed by Lee et al., found a minimum error of −38% and a maximum error of 26% between automatically determined portion weights and control weights of 19 individual foods [20,24]. Nonetheless, the relatively small number of participants and items assessed in the aforementioned studies reduce the likelihood of extreme errors compared to the present study.

MFR performance was particularly challenged by small or hidden ingredients within records. The greatest mean absolute weight errors were observed in the “Fats & oils”, “Sweeteners” and “Condiments & sauces” food groups. In the linear regression, the 95% limits of agreement for alcohol extended into the negatives, likely reflecting the oversight of alcohol in sauces and the misclassification of alcoholic beverages by MFR. Imperceptible elements (e.g., sugar, oils and sauces) were indeed harder to classify by the app and showed weaker classification sensitivity compared to other food groups, an inevitable limitation of dietary data collection by photography [24]. The same conclusion can be extended to segmentation, where omissions and intrusions made by MFR mainly affected subsidiary food items, such as capers, sauces, or vinegar, as well as two additional segments blended in vegetable mixes.

The segmentation, classification and portion size estimation findings all influence the overall performance of MFR. We observed higher coefficients of variation Cυ for energy and macronutrient estimates when true quantities were small, with a tendency towards overestimation. After a certain threshold, MFR underestimated all macronutrients with the exception of proteins. MFR’s overestimation tendency towards proteins could be exacerbated by the significant weight overestimation of segments of “Meat & Poultry”, “Fish & Seafood” as well as “Eggs and Meat substitutes”. While carbohydrate estimates of all records showed reasonable results ($\bar{C\upsilon }$ = 0.31), fiber and alcohol had the highest mean coefficient of dispersion globally (0.58 and 1.25, respectively), especially in the case of beverages. Overall, linear regression analysis showed wide limits of agreements between MFR and weight record control method for the energy, fat, carbohydrates, proteins, fiber and alcohol content of all records. Wide limits of agreement between a novel method and a control method are commonly observed in similar studies, whose digital dietary assessment tools are often validated for a utilization at the group level [9,21,22].

Nonetheless, our methodology focused on MFR’s performance as a dietary assessment device, with no consideration regarding true daily dietary intake and real-life conditions (i.e., study participants taking pictures of their food on selected days). This made the comparison with other digital tools difficult and restricted our analysis to a specific record’s energy and macronutrient accuracy and precision. To avoid discriminating against MFR for erroneously classifying or forgetting segments, we assessed weight errors on exactly classified segments only, which constitutes another limitation of our work. Furthermore, the decision not to use the app’s comment fields during data collection may have reduced the accuracy of MFR. Indeed, MFR users are normally able to provide spontaneous description or comments in these integrated annotation fields, but we intentionally ignored this tool in the present study, in order to test MFR’s sole capability to identify and classify record content.

## 5. Conclusions and Recommendations

In light of the above, we would advise caution in the analysis of energy and macronutrient content for precise individual dietary assessment. Good agreement for portion size estimation between MFR and weighted food diaries, along with the app’s strong segmentation and classification capabilities appears to be nonetheless suited for the identification of dietary patterns, eating habits and regime types.

Statistical recalibrations to adjust for measurement error could potentially be used to improve MFR’s current estimations. Energy adjustments could also be applied to increase the overall accuracy of MFR. This analytic method, which helps mitigate the effects of measurement errors when data are collected via a self-reported dietary assessment tool, has been assessed and applied in similar validation studies and could constitute the subject of subsequent research, provided that total energy intake is assessed [22,40].

Currently, MFR’s energy and macronutrient assessment is highly affected by imprecise portion size estimation. Improving portion size estimation capabilities would therefore prove valuable in strengthening the app’s general performance. Combined with MFR’s user-friendly recording interface, this would distinguish the app from other digital dietary assessment tools currently available for research purposes. Supported by a significant classification improvement with annotators’ intervention, we would recommend MFR developers to focus on beverage content identification, to enhance MFR classification accuracy. The presence of alcohol, milk or sugar in beverages should be of particular focus and could be flagged by systematically asking participants for the content of their beverages. This is, for instance, applied in the mobile device food record mpFR, which allows users to rectify misclassified segments before confirmation of intake [41,42]. MFR already features an optional field for remarks which is visible during record entry. It would be in the app user’s best interest to benefit from systematic prompts to ensure a more accurate classification of composite beverages. The same recommendation could be made for sauces and condiments.

These adaptations could be put to the test in a subsequent study, further investigating MFR use in real-life settings with the measurement of daily dietary intake from study participants. In such conditions, and in order to fully compare MFR performance and practical implementation in epidemiological studies over traditional dietary assessment methods, researchers should assess the relevance of participants’ notes, potential prompts or the use of a fiduciary marker on the pictures for portion size estimation and energy and macronutrient calculation. Tradeoffs in terms of time, cost-efficiency and practicability should nevertheless be considered to avoid increasing user burden.

## Figures and Tables

**Table 1:** Segmentation accuracy: proportion ± standard deviation (and number of segments) found, omitted and intruded by MFR, by record type.

Segments	Total (*n* = 352)	Composite Foods (*n* = 208)	Simple Foods (*n* = 79)	Composite Beverages (*n* = 32)	Simple Beverages (*n* = 33)
%Found (*n*)	98.0% ± 1.5 (*n* = 345)	97.6% ± 2.1 (*n* = 203)	98.7% ± 2.5 (*n* = 78)	96.9% ± 6.1 (*n* = 31)	100.0% ± 0.0 (*n* = 33)
%Omitted (*n*)	2.0% ± 1.5 (*n* = 7)	2.4% ± 2.1 (*n* = 5)	1.3% ± 2.5 (*n* = 1)	3.1% ± 6.1 (*n* = 1)	0.0% (*n* = 0)
%Intruded (*n*)	1.4% ± 1.2 (*n* = 5)	1.5% ± 1.7 (*n* = 3)	2.5% ± 3.5 (*n* = 2)	0.0% (*n* = 0)	0.0% (*n* = 0)

**Table 2:** Classification accuracy: proportion ± standard deviation (and number of found segments) classified as exact match, close match, far match and mismatch, by record type.

Records Classification	Total (*n* = 345)	Composite Foods (*n* = 203)	Simple Foods (*n* = 78)	Composite Beverages (*n* = 31)	Simple Beverages (*n* = 33)
%Exact match (*n*)	87.5% ± 3.5 (*n* = 302)	90.1% ± 4.1 (*n* = 183)	96.2% ± 4.3 (*n* = 75)	41.9% ± 17.7 (*n* = 13)	93.9%± 8.3 (*n* = 31)
%Close match (*n*)	8.4% ± 3.0 (*n* = 29)	6.9% ± 3.5 (*n* = 14)	3.8% ± 4.3 (*n* = 3)	38.7% ± 17.4 (*n* = 12)	0.0% (*n* = 0)
%Far match (*n*)	1.2% ± 1.1 (*n* = 4)	2.0% ± 1.9 (*n* = 4)	0.0% (*n* = 0)	0.0% (*n* = 0)	0.0% (*n* = 0)
%Mismatch (*n*)	2.9% ± 1.8 (*n* = 10)	1.0% ± 1.4 (*n* = 2)	0.0% (*n* = 0)	19.4% ± 14.1 (*n* = 6)	6.1% ± 8.3 (*n* = 2)

**Table 3:** Classification accuracy: Cohen’s kappa, uniform kappa, sensitivity and specificity of MFR classification compared to controlled values from weighted food diaries, by food groups. (NaNs: non-alcoholic non-sweetened; NaS: Non-alcoholic sweetened).

Food Groups	Cohen Kappa		Uniform Kappa		Sensitivity [%]		Specificity [%]	
	Kappa	Std. Err.	Kappa	[95% CI]	Sensitivity	[95% CI]	Specificity	[95% CI]
NaNs beverages	0.8607	0.0533	0.977	[0.954;0.994]	100	[75.3;100]	98.8	[96.9; 99.7]
Vegetables	1.0000	0.0538	1	[1;1]	100	[95.3;100]	100	[98.6;100]
Fruit	1.0000	0.0538	1	[1;1]	100	[85.8;100]	100	[98.9;100]
Juice	0.7721	0.0524	0.977	[0.954;0.994]	100	[59;100]	98.8	[97.0;99.7]
Meat & poultry	1.0000	0.0538	1	[1;1]	100	[83.9;100]	100	[98.9;100]
Fish & seafood	1.0000	0.0538	1	[1;1]	100	[54.1;100]	100	[98.9;100]
Unclassified meat	1.0000	0.0538	1	[1;1]	100	[39.8;100]	100	[98.9;100]
Eggs & meat substitutes	0.8874	0.0535	0.994	[0.983;1]	100	[39.8;100]	99.7	[98.4;100]
Dairy products (excl. milk)	1.0000	0.0538	1	[1;1]	100	[80.5;100]	100	[98.9;100]
Milk & milk-based beverages	1.0000	0.0538	1	[1;1]	100	[29.2;100]	100	[98.9,100]
Seeds & nuts	1.0000	0.0538	1	[1;1]	100	[29.2;100]	100	[98.9;100]
Fats & oils	0.8542	0.0538	0.988	[0.971;1]	85.7	[42.1;99.6]	99.7	[98.4;100]
Cereals & cereal-based products	0.9598	0.0538	0.988	[0.971;1]	96.3	[81; 99.9]	99.7	[98.3;100]
Rice, rice-based products	0.9319	0.0537	0.994	[0.983;1]	100	[59;100]	99.7	[98.4;100]
Potatoes, legumes & beans	0.9469	0.0538	0.988	[0.971;1]	90.5	[69.6;98.8]	100	[98.9;100]
Salty snacks	1.0000	0.0538	1	[1;1]	100	[54.1;100]	100	[98.9;100]
Sweet dishes	1.0000	0.0538	1	[1;1]	100	[78.2;100]	100	[98.9;100]
Sweeteners	0.9076	0.0536	0.994	[0.983;1]	83.3	[35.9;99.6]	100	[98.9;100]
NaS beverages	0.8122	0.0536	0.977	[0.954;0.994]	75.0	[42.8;94.5]	99.7	[98.3;100]
Alcoholic beverages	0.8574	0.0533	0.971	[0.936;0.994]	76.2	[52.8;91.8]	100	[98.9;100]
Condiments & sauces	0.9665	0.0538	0.988	[0.971;1]	97.0	[84.2;99.9]	99.7	[98.2;100]
Milk substitutes	1.0000	0.0538	1	[1;1]	100	[2.5;100]	100	[98.9;100]
Soups	1.0000	0.0538	1	[1;1]	100	[69.2;100]	100	[98.9;100]

**Table 4:** Global classification reliability (Cohen kappa) and agreement (uniform kappa) between MyFoodRepo and controlled values from weighted food diaries.

Level of Granularity	Cohen Kappa		Uniform Kappa	
	Kappa	Std. Err.	Kappa	[95% CI]
Food Categories	0.9603	0.0254	0.963	[0.943; 0.983]
Food Groups	0.9554	0.0158	0.958	[0.933; 0.979]
Food Types	0.9559	0.0145	0.958	[0.934; 0.979]

**Table 5:** Overall performance for energy and macronutrient content: Coefficient of variation and mean coefficient of variation of all MFR estimates calculated at the 25th percentile, median and 75th percentile of controlled values for energy, fat, carbohydrates, fiber and alcohol.

	Coefficient of Variation Cυ			Mean Coefficient of Variation $\bar{C\upsilon }$
	At True Values’ 25th Percentile	At True Values’ Median	At True Values’ 75th Percentile	All Records
Energy	0.58	0.45	0.37	0.35
Fat	1.68	0.83	0.52	0.42
Carbohydrates	0.65	0.45	0.37	0.31
Protein	1.96	0.63	0.40	0.38
Fiber	1.47	0.72	0.62	0.58
Alcohol	1.70	1.70	1.70	1.25

**Table 6:** Overall performance for energy and macronutrient content by record type: Mean coefficient of variation of MFR estimates for energy, fat, carbohydrates, fibers and alcohol by record type.

	Mean Coefficient of Variation $\bar{C\upsilon }$		
	All Records	Foods	Beverages
Energy	0.35	0.33	0.75
Fat	0.42	0.41	1.18
Carbohydrates	0.31	0.32	0.27
Protein	0.38	0.37	0.65
Fibers	0.58	0.52	2.01
Alcohol	1.25	3.14	1.23

## Data Availability

The data presented in this study are available on request from the corresponding author. The data are not publicly available due to intellectual property reasons.

## Supplementary Materials

The following are available online at https://www.mdpi.com/article/10.3390/nu14030635/s1, Table S1: Description of food types and corresponding food groups and food categories, Figure S1: Characteristics of records collected, Table S2: Cohen kappa, uniform kappa, sensitivity and specificity of MFR classification compared to controlled values from weighted food diaries, by food types, Table S3: Cohen kappa, uniform kappa, sensitivity and specificity of MFR classification compared to controlled values from weighted food diaries, by food categories, Table S4: MFR portion size estimation performance: mean estimated weights versus true weight, mean error and mean absolute error of all exactly classified segments, displayed by food types, Table S5: MFR portion size estimation performance: mean estimated weights versus true weights, mean error and mean absolute error of all exactly classified segments, displayed by food groups, Table S6: MFR portion size estimation performance: mean estimated weights versus true weights, mean error and mean absolute error of all exactly classified segments, displayed by food categories, Figure S2: Linear regression of MFR versus controlled values for all found food records, for content of (a) energy; (b) fat; (c) carbohydrates; (d) protein; (e) fiber and (f) alcohol, Figure S3: Linear regression of MFR versus controlled values for all found beverage records, for content of (a) energy; (b) fat; (c) carbohydrates; (d) protein; (e) fiber and (f) alcohol.

## References

[1] Thompson F.E., Subar A.F., Coulston A.M., Boushey C.J., Ferruzzi M. (2013). Dietary assessment methodology. Nutrition in the Prevention and Treatment of Disease.

[2] Blanchard C.M., Chin M.K., Gilhooly C.H., Barger K., Matuszek G., Miki A.J., Côté R.G., Eldridge A.L., Green H., Mainardi F. (2021). Evaluation of PIQNIQ, a Novel Mobile Application for Capturing Dietary Intake. J. Nutr..

[3] Evenepoel C., Clevers E., Deroover L., Van Loo W., Matthys C., Verbeke K. (2020). Accuracy of Nutrient Calculations Using the Consumer-Focused Online App MyFitnessPal: Validation Study. J. Med. Internet Res..

[4] Illner A.K., Freisling H., Boeing H., Huybrechts I., Crispim S.P., Slimani N. (2012). Review and evaluation of innovative technologies for measuring diet in nutritional epidemiology. Int. J. Epidemiol..

[5] Sharp D.B., Allman-Farinelli M. (2014). Feasibility and validity of mobile phones to assess dietary intake. Nutrition.

[6] Research2Guidance (2018). mHealth Developer Economics—Connectivity in Digital Health.

[7] Chen J., Cade J.E., Allman-Farinelli M. (2015). The Most Popular Smartphone Apps for Weight Loss: A Quality Assessment. JMIR mhealth uhealth.

[8] Griffiths C., Harnack L., Pereira M.A. (2018). Assessment of the accuracy of nutrient calculations of five popular nutrition tracking applications. Public Health Nutr..

[9] Ji Y., Plourde H., Bouzo V., Kilgour R.D., Cohen T.R. (2020). Validity and Usability of a Smartphone Image-Based Dietary Assessment App Compared to 3-Day Food Diaries in Assessing Dietary Intake Among Canadian Adults: Randomized Controlled Trial. JMIR mHealth uHealth.

[10] Shinozaki N., Murakami K. (2020). Evaluation of the Ability of Diet-Tracking Mobile Applications to Estimate Energy and Nutrient Intake in Japan. Nutrients.

[11] Marcano-Olivier M., Erjavec M., Horne P.J., Viktor S., Pearson R. (2019). Measuring lunchtime consumption in school cafeterias: A validation study of the use of digital photography. Public Health Nutr..

[12] Gemming L., Rush E., Maddison R., Doherty A., Gant N., Utter J., Ni Mhurchu C. (2015). Wearable cameras can reduce dietary under-reporting: Doubly labelled water validation of a camera-assisted 24 h recall. Br. J. Nutr..

[13] Jia W., Chen H.C., Yue Y., Li Z., Fernstrom J., Bai Y., Li C., Sun M. (2014). Accuracy of food portion size estimation from digital pictures acquired by a chest-worn camera. Public Health Nutr..

[14] Pettitt C., Liu J., Kwasnicki R.M., Yang G.Z., Preston T., Frost G. (2016). A pilot study to determine whether using a lightweight, wearable micro-camera improves dietary assessment accuracy and offers information on macronutrients and eating rate. Br. J. Nutr..

[15] Ahmad Z., Kerr D.A., Bosch M., Boushey C.J., Delp E.J., Khanna N., Zhu F. (2016). A Mobile Food Record For Integrated Dietary Assessment. MADiMa16.

[16] Casperson S.L., Sieling J., Moon J., Johnson L., Roemmich J.N., Whigham L. (2015). A mobile phone food record app to digitally capture dietary intake for adolescents in a free-living environment: Usability study. JMIR mHealth uHealth.

[17] Rhyner D., Loher H., Dehais J., Anthimopoulos M., Shevchik S., Botwey R.H., Duke D., Stettler C., Diem P., Mougiakakou S. (2016). Carbohydrate Estimation by a Mobile Phone-Based System Versus Self-Estimations of Individuals With Type 1 Diabetes Mellitus: A Comparative Study. J. Med. Internet Res..

[18] He H., Kong F., Tan J. (2016). DietCam: Multiview Food Recognition Using a Multikernel SVM. IEEE J. Biomed. Health Inform..

[19] Bucher Della Torre S., Carrard I., Farina E., Danuser B., Kruseman M. (2017). Development and Evaluation of e-CA, an Electronic Mobile-Based Food Record. Nutrients.

[20] Lee C.D., Chae J., Schap T.E., Kerr D.A., Delp E.J., Ebert D.S., Boushey C.J. (2012). Comparison of known food weights with image-based portion-size automated estimation and adolescents’ self-reported portion size. J. Diabetes Sci. Technol..

[21] Lemacks J.L., Adams K., Lovetere A. (2019). Dietary Intake Reporting Accuracy of the Bridge2U Mobile Application Food Log Compared to Control Meal and Dietary Recall Methods. Nutrients.

[22] Wellard-Cole L., Chen J., Davies A., Wong A., Huynh S., Rangan A., Allman-Farinelli M. (2019). Relative Validity of the Eat and Track (EaT) Smartphone App for Collection of Dietary Intake Data in 18-to-30-Year Olds. Nutrients.

[23] Zhang W., Yu Q., Siddiquie B., Divakaran A., Sawhney H. (2015). “Snap-n-Eat”: Food Recognition and Nutrition Estimation on a Smartphone. J. Diabetes Sci. Technol..

[24] Höchsmann C., Martin C.K. (2020). Review of the validity and feasibility of image-assisted methods for dietary assessment. Int. J. Obes..

[25] Digital Epidemiology Lab. The app MyFoodRepo. https://www.foodandyou.ch/en/my-food-repo.

[26] Office Fédéral de la Securité Alimentaire et des Affaires Vétérinaires. Base de Données Suisse des Valeurs Nutritives. https://valeursnutritives.ch/fr/.

[27] Agence Nationale de Sécurité Sanitaire de L’alimentation de L’environnement et du Travail (Anses). Table de Composition Nutritionnelle des Aliments CIQUAL. https://ciqual.anses.fr/.

[28] Max Rubner-Institut (2020). German Nutrient Database (Bundeslebensmittelschlüssel, BLS).

[29] Société Suisse de Nutrition SSN, Office Fédéral de la Securité Alimentaire et des Affaires Vétérinaires (2011). La Pyramide Alimentaire Suisse.

[30] Laboratoire D’epidémiologie Numérique EPFL. Open Food Repo. www.foodrepo.org.

[31] Brennan R.L., Prediger D.J. (1981). Coefficient Kappa: Some Uses, Misuses, and Alternatives. Educ. Psychol. Meas..

[32] Taffe P., Zuppinger C., Burger G.M., Gonseth-Nusslé S. (2022). The Bland-Altman Method Should Not Be Used When One of the Two Measurement Methods Has Negligible Measurement Errors.

[33] Taffe P. (2018). Effective plots to assess bias and precision in method comparison studies. Stat. Methods Med. Res..

[34] Martin C.K., Kaya S., Gunturk B.K. Quantification of food intake using food image analysis. Proceedings of the 2009 Annual International Conference of the IEEE Engineering in Medicine and Biology Society.

[35] Martin C.K., Correa J.B., Han H., Allen H.R., Rood J.C., Champagne C.M., Gunturk B.K., Bray G.A. (2012). Validity of the Remote Food Photography Method (RFPM) for estimating energy and nutrient intake in near real-time. Obesity.

[36] Daugherty B.L., Schap T.E., Ettienne-Gittens R., Zhu F.M., Bosch M., Delp E.J., Ebert D.S., Kerr D.A., Boushey C.J. (2012). Novel technologies for assessing dietary intake: Evaluating the usability of a mobile telephone food record among adults and adolescents. J. Med. Internet Res..

[37] Harnack L., Steffen L., Arnett D.K., Gao S., Luepker R.V. (2004). Accuracy of estimation of large food portions. J. Am. Diet. Assoc..

[38] Flax V.L., Thakwalakwa C., Schnefke C.H., Stobaugh H., Phuka J.C., Coates J., Rogers B., Bell W., Colaiezzi B., Muth M.K. (2019). Validation of a digitally displayed photographic food portion-size estimation aid among women in urban and rural Malawi. Public Health Nutr..

[39] Norman Å., Kjellenberg K., Torres Aréchiga D., Löf M., Patterson E. (2020). “Everyone can take photos”. Feasibility and relative validity of phone photography-based assessment of children’s diets—A mixed methods study. Nutr. J..

[40] Subar A.F., Freedman L.S., Tooze J.A., Kirkpatrick S.I., Boushey C., Neuhouser M.L., Thompson F.E., Potischman N., Guenther P.M., Tarasuk V. (2015). Addressing Current Criticism Regarding the Value of Self-Report Dietary Data. J. Nutr..

[41] Khanna N., Boushey C.J., Kerr D., Okos M., Ebert D.S., Delp E.J. An Overview of The Technology Assisted Dietary Assessment Project at Purdue University. Proceedings of the 2010 IEEE International Symposium on Multimedia.

[42] Woo I., Otsmo K., Kim S., Ebert D.S., Delp E.J., Boushey C.J. Automatic portion estimation and visual refinement in mobile dietary assessment. Proceedings of the IS&T/SPIE Electronic Imaging.

